# Molecular epidemiology and carbapenem resistance mechanisms of *Pseudomonas aeruginosa* isolated from a hospital in Fujian, China

**DOI:** 10.3389/fmicb.2024.1431154

**Published:** 2024-09-05

**Authors:** Xueqin Xie, Zhou Liu, Jingyan Huang, Xueting Wang, Yuting Tian, Pinying Xu, Gangsen Zheng

**Affiliations:** ^1^Department of Basic Medical Science, Xiamen Medical College, Xiamen, China; ^2^Provincial Key Laboratory of Functional and Clinical Translational Medicine of Universities in Fujian, Xiamen Medical College, Xiamen, China; ^3^Institute of Respiratory Disease, Xiamen Medical College, Xiamen, China; ^4^Xiamen Key Laboratory of Genetic Testing, Department of Laboratory Medicine, The First Affiliated Hospital of Xiamen University, School of Medicine, Xiamen University, Xiamen, China

**Keywords:** *Pseudomonas aeruginosa*, multilocus sequence typing, carbapenemase, porins mutation, biofilm, multidrug efflux pump genes

## Abstract

The worldwide spread of *Pseudomonas aeruginosa*, especially carbapenem-resistant *P*. *aeruginosa* (CRPA), poses a serious threat to global public health. In this research, we collected and studied the clinical prevalence, molecular epidemiology, and resistance mechanisms of CRPA in Fujian, China. Among 167 non-duplicated *P*. *aeruginosa* isolates collected during 2019–2021, strains from respiratory specimens and wound secretions of older males in the intensive care unit dominated. Ninety-eight isolates (58.7 %) were resistant to at least one tested antibiotic, among which 70 strains were carbapenem-resistant. Moleclar typing of the CRPA isolates revealed they were highly divergent, belonging to 46 different sequence types. It is noteworthy that two previously reported high risk clones, ST1971 specific to China and the globally prevalent ST357, were found. Several carbapenem resistance-related characteristics were also explored in 70 CRPA isolates. Firstly, carbapenemase was phenotypically positive in 22.9 % of CRPA, genetically predominant by metallo-β-lactamase (MBL) and co-carrige of different carbapenemase genes. Then, mutations of the carbapenem-specific porins oprD and opdP were commonly observed, with frequencies of 97.1% and 100.0%, respectively. Furthermore, the biofilm formation and relative transcription levels of 8 multidrug efflux pump genes were also found to be increased in 48.6 % and 72.9 % of CRPA isolates compared to the reference strain PAO1. These findings will help fill the data gaps in molecular characteristics of CRPA on the southeastern coast of China and emphasize the urgent need for data-based specific stewardship for antipseudomonal practices to prevent the dissemination of CRPA.

## Introduction

*Pseudomonas aeruginosa* (PA) is widely distributed and is considered as one of the most common opportunistic pathogens responsible for hospital-acquired infections (HAIs) (Qin et al., 2022). In recent years, the emerging and worldwide spread of multidrug-resistant (MDR)/extensively drug-resistant (XDR) ‘high-risk clones', especially carbapenem-resistant *P*. *aeruginosa* (CRPA), have further worsened patient outcomes for nosocomial infections of this pathogen (Karruli et al., 2023).

CRPA has been ranked by the World Health Organization (WHO) as a pathogen of highest priority in the “critical” category, urgently requiring novel antibiotics in clinical settings [World Health Organization (WHO) (2017)]. The global increase in CRPA incidence is particularly concerning due to the lack of effective treatment alternative. Various mechanisms, alone or combined, including mutation leading to an impermeable outer membrane (Atrissi et al., 2021), production of carbapenemase (Tenover et al., 2022), overexpression of efflux systems (Kao et al., 2016), and formation of biofilm (Ma et al., 2023), have been reported to be commonly found in CRPA. Among them, the carbapenemase-mediated mechanism is an increasing contributor of significant concern worldwide (Wang et al., 2021; Tenover et al., 2022). Firstly, the emergence of carbapenemase greatly weakens the efficacy of both commonly used antipseudomonal agents and newly introduced β-lactam/β-lactamase inhibitor combinations (Canton et al., 2022; Tenover et al., 2022). Furthermore, the carbapenem-resistant determinants in these organisms are usually carried by mobile genetic elements and frequently harbor additional resistance determinants (Yoon and Jeong, 2021). Thus, they not only greatly limit the choice of anti-infective strategies but also enhance the dissemination risk of resistant clones.

However, the carbapenemase enzymogram present in *P*. *aeruginosa* varies widely by region, including the Ambler Class A β-lactamases, metallo-β-lactamases (MBL), and the Class D enzymes (Hammoudi Halat and Ayoub Moubareck, 2022). Rapid confirmation and differentiation among the various classes of carbapenemases are crucial for initiating early and effective therapy. Local surveillance and prompt profiling of the predominant carbapenemase phenotype and genotype will facilitate data-driven precise therapeutic decisions and thus positive outcomes. In this paper, we analyze the clinical prevalence, molecular epidemiology and carbapenem resistance-ralated characteristics of clinical *P*. *aeruginosa*, particularly CRPA isolates, collected over three consecutive years in a representative hospital in Xiamen, Fujian, in southeast China. This study aims to enhance our understanding of their prevalence and thus facilitate developing effective control strategies.

## Materials and methods

### Bacterial isolates

A total of 167 non-duplicate (one strain maximum per patient) clinical isolates of *P*. *aeruginosa* were collected at the First Affiliated Hospital of Xiamen University from April 2019 to June 2021. Strains were isolated from various clinical specimens and identified using MALDI-TOF MS (Bruker Daltonics, Germany). *P*. *aeruginosa* PAO1 (Grace et al., 2022) was used as a reference strain in porin mutation analysis, quantification of biofilm production and relative transcription of efflux pump genes.

### Antimicrobial susceptibility test of all collected isolates

The minimum inhibitory concentrations (MICs) of 167 clinical PA isolates against 13 antibacterial drugs from 7 different categories were determined using the broth microdilution method following the guidelines of the Clinical and Laboratory Standards Institute (CLSI), with *P*. *aeruginosa* ATCC 27853 as the quality control strain [Clinical and Laboratory Standards Institute (CLSI), 2022]. The antimicrobials tested included aminoglycosides (amikacin/AMK, gentamicin/GEN, and tobramycin/TOB), cephalosporins (ceftazidime/CAZ and cefepime/FEP), carbapenems (imipenem/IMP and meropenem/MEM), quinolones (ciprofloxacin/CIP and levofloxacin/LVX), β-lactams/β-lactamase inhibitors (piperacillin/tazobactam/TZP and ticarcillin/clavulanic acid/TCC), monobactam (aztreonam/ATM), and penicillin (piperacillin/PIP). MDR isolates were defined as those resistant to at least one agent in three or more antimicrobial categories (Magiorakos et al., 2012). The CRPA isolates were defined as strains resistant to either imipenem or meropenem, or both, with a MIC value of ≥8 μg/mL.

### Multilocus sequence typing (MLST) of CRPA

MLST analysis of 70 CRPA isolates was performed as described in the PubMLST database of *P*. *aeruginosa* (Jolley et al., 2018) through polymerase chain reaction (PCR) amplification and sequencing of seven housekeeping genes (*acsA, aroE, guaA, mutL, nuoD, ppsA*, and *trpE*). Gene sequences were then submitted to query the PubMLST database (http://pubmlst.org/paeruginosa/) to determine the allelic numbers and sequence types (STs). The types that could not match any known types were submitted to obtain new STs. A minimum spanning tree (MST) was inferred using the goeBURST algorithm (http://www.phyloviz.net/) (Ribeiro-Gonçalves et al., 2016) based on the MLST allelic profiles. A clonal complex (CC) is defined as containing at least two STs sharing any six out of the seven alleles.

### Carbapenem resistance mechanisms of CRPA

#### Phenotypic and genotypic carbapenemase testing of CRPA

Testing and interpreting of phenotypic carbapenemase in 70 CRPA isolates were performed by modified carbapenem inactivation method (mCIM) per CLSI standard [Clinical and Laboratory Standards Institute (CLSI), 2022]. Routine quality control was conducted with each mCIM run with carbapenemase-positive *Klebsiella pneumoniae* ATCC BAA-1705 and carbapenemase-negative *K*. *pneumoniae* ATCC BAA-1706. Additionally, meropenem disks incubated in tryptic soy broth (TSB) with no microbial inocula for 4 h at 37°C were also applied to the *Escherichia coli* ATCC 25922 lawn. Zones were evaluated after overnight incubation to ensure they fell within CLSI quality control ranges.

All mCIM positive isolates were then assessed for the presence of carbapenemase genes through PCR. Nine carbapenemase genes, including *bla*_KPC_, *bla*_GES_, *bla*_IMP_, *bla*_NDM_, *bla*_VIM_, *bla*_OXA − 23 − like_, *bla*_OXA − 24 − like_, *bla*_OXA − 48 − like_ and *bla*_OXA − 58 − like_, were amplified using the primers presented in Table 1. The positive amplification products were sequenced and compared with those available in the GenBank database (www.ncbi.nil.gov/BLAST) to identify specific variant.

**Table 1:** Primers used in this study.

**Aims**	**Genes**	**Primers (5^′^-3^′^)**	**Amplicon (bp)**	**References**
Carbapenemase genotyping by PCR	*bla*_KPC_	KPC-F/R: TGTCACTGTATCGCCGTC/ TCAGTGCTCTACAGAAAACC	1111	Khan et al. (2017)
	*bla*_GES_	GES-F/R: ATGCGCTTCATTCACGCAC/ CTATTTGTCCGTGCTCAGG	864	Poirel et al. (2000)
	*bla*_IMP_	IMP-F/R: GGAATAGAGTGGCTTAAYTCTC/ GGTTTAAYAAAACAACCACC	232	Poirel et al. (2011)
	*bla*_NDM_	NDM-F/R: TGGAATTGCCCAATATTATGC/ TCAGCGCAGCTTGTCGGCCATGCG	813	Nordmann et al. (2012)
	*bla*_VIM_	VIM-F/R: GATGGTGTTTGGTCGCATA/ CGAATGCGCAGCACCAG	400	This study
	*bla*_OXA − 23 − like_	OXA-23-F/R: GATCGGATTGGAGAACCAGA/ ATTTCTGACCGCATTTCCAT	501	Woodford et al. (2006)
	*bla*_OXA − 24 − like_	OXA-24-F/R: GGTTAGTTGGCCCCCTTAAA/ AGTTGAGCGAAAAGGGGATT	249	Woodford et al. (2006)
	*bla*_OXA − 48 − like_	OXA-48-F/R: GCGTGGTTAAGGATGAACAC/ CATCAAGTTCAACCCAACCG	438	Poirel et al. (2011)
	*bla*_OXA − 58 − like_	OXA-58-F/R:AAGTATTGGGGCTTGTGCTG/ CCCCTCTGCGCTCTACATAC	599	Woodford et al. (2006)
Porin mutation analysis by PCR	*oprD*	OprD-F/R: CGCCGACAAGAAGAACTAGC/ CGGTACCTACGCCCTTCCTT	1496	Yin et al. (2018)
	*opdP*	OpdP-F/R: CCGGCGCAGGCGAAACCGGCGC/ CAGGTGTCGGAGCAACGGATGG	1604	This study
Quantitative real-time PCR (qRT-PCR) of multidrug efflux pump genes	*MexA*	MexA-RT-F/R: AGCCATGCGTGTACTGGTTC/ CTCGGTATTCAGGGTCACCG	145	This study
	*MexB*	MexB-RT-F/R: CTGTCGATCCTCAGTCTGCC/ TCGATCCCGTTCATCTGCTG	146	This study
	*MexC*	MexC-RT-F/R: TACCGGCGTCATGCAGGGTTC/ TTACTGTTGCGGCGCAGGTGACT	164	This study
	*MexD*	MexD-RT-F/R: AAGCGTGCTCGAGCTATACG/ CCCTCTTCCCATTTCACGCT	84	This study
	*MexE*	MexE-RT-F/R: CTGAGCTTCACCCGGATCAC/ CGTCGAAGTAGGCGTAGACC	139	This study
	*MexF*	MexF-RT-F/R: GCTTCGGCCGTACCTATCAG/ AGGTGTCGCTGACCTTGATG	141	This study
	*MexX*	MexX-RT-F/R: GAAGGCCAGGTGAAGGGTG/ CCAGGTCGGAGAACAGCAG	106	This study
	*MexY*	MexY-RT-F/R: TCGCCGTGATGTACCTGTTC/ ACGTTGATCGAGAAGCCCAG	121	This study
	*RspL*	rspL-140-F/R: CACAACCTGCAAGAGCACAG/ CCGTACTTCGAACGACCCTG	140	This study

#### Analysis of oprD and opdP mutations in CRPA

The full-length *oprD* and *opdP* genes from each CRPA isolate were amplified and sequenced using the primers listed in Table 1. DNA sequences and protein transcripts were compared with the corresponding sequences from the reference strain PAO1 (accession number NC_002516) using MEGA11 to dentify mutations (Tamura et al., 2021).

#### qRT-PCR assay for transcriptional levels of multidrug efflux pump genes in CRPA

To evaluate the correlation of multidrug efflux system with carbapenem resistance, the relative transcriptional levels of 8 Mex transporter genes (Table 1) in all 70 CRPA isolates were determined against PAO1 using qRT-PCR. Total RNA of these strains was extracted and purified using the RNAsimple Total RNA Kit (Tiangen Biotech, Beijing, China) according to the manufacturer's instructions. The cDNA was synthesized using the Hifair^®^ V one-step RT-gDNA digestion SuperMix for qPCR (Yeasen, Shanghai, China). qRT-PCR was performed on the QuantStudio 5 real-time PCR system (Applied Biosystems) with SYBR Green Master Mix (Yeasen, Shanghai, China). The housekeeping gene *rspL* was used for normalization of the expression levels of the target genes. Each test was performed in triplicates and the results were analyzed by the QuantStudio Design & Analysis Software 2.6.0.

#### Assay for biofilm formation of CRPA

The biofilm formed by 70 CRPA isolates was quantified using microtiter plate assay (Haney et al., 2021). Briefly, the isolates were grown overnight in LB at 37°C and then adjusted to an OD_600_ = 0.1. Twenty microliters of each culture were inoculated to 180 μL LB in 96-well plates, and incubated at 37°C in a static incubator for 24h. Eight replicates were tested for each isolates and negative control wells contained LB only. After measuring the OD_600_ to evaluate planktonic growth, the bacteria were discarded and each well was washed three times with 250 μL PBS, dried for 30 min at room temperature, and then stained with 250 μL of 0.1 % crystal violet for 40 min. After rinsing off excess stains by washing three times with PBS, the plate was dried for another 30 min. The biofilm was resuspended with 260 μL of 95 % ethanol and quantified at 590 nm using a SpectraMax_i3X microplate spectrophotometer (Molecular Devices, Austria).

### Statistical analysis

Data were analyzed with the GraphPad Prism analysis package. The antimicrobial rates of PA in different groups was compared using Pearson's chi-square test. One-way ANOVA (Analysis of Variance) analysis with Holm-Sidak comparison test was used to determine statistical differences of biofilm formation and relative transcription level of efflux genes between groups. A Spearman correlation test was performed to analyze the association of carbapenem resistance (indicated with MIC values) with possible mechanisms (i.e., carbapenemase production, porin mutation, relative transcription of multidrug pump genes and biofilm yield). *P* < 0.05 was considered statistically significant.

Moreover, an overall flowchart of the research (Supplementary Figure S1) was presented.

## Results

### Clinical prevalence of all collected *P*. *aeruginosa* isolates

#### Isolation sources, specimen types, and demographics of patients

A total of 167 non-duplicate PA isolates were collected from samples across 26 different clinical departments (Table 2). They were most commonly isolated from the intensive care unit (ICU) followed by the geriatric medicine department. Varying numbers (*n* = 1–11) of PA strains were also isolated in departments such as orthopedics, hepatobiliary and pancreatic vascular surgery, hematology, and so on.

**Table 2:** The prevalent characteristics of clinical *Pseudomonas aeruginosa* (PA) isolates.

**Prevalent characteristics of clinical PA isolates**	**Means ±SD (range) or *n* (%)**
**Isolation sources**	
ICU	38 (22.8 %)
Geriatric medicine department	23 (13.8 %)
Orthopedics	11 (6.6 %)
Hepatobiliary and pancreatic vascular surgery	10 (6.0 %)
Hematology	10 (6.0 %)
Others (21 different departments)	75 (44.9 %)
**Specimen types**	
Respiratory samples	43 (25.7 %)
Wound secretions	43 (25.7 %)
Puncture fluids^a^	30 (18.0 %)
Blood	23 (13.8 %)
Urine	11 (6.6 %)
Gastrointestinal tract samples	7 (4.2 %)
Others (6 different specimen types)	10 (6.0 %)
**Demographics of patients**	
Age, years	61 ± 22 (1–94)
Gender	
Female	45 (26.9 %)
Male	122 (73.1 %)

^a^The puncture fluids there included cerebral spinal fluid, bile, pleural effusion, ascites and synovial fluid.

The respiratory samples and wound secretions represented as the most common specimen. Puncture fluids and blood were also frequently encountered, with more than 20 isolates collected each. The remaining 28 PA strains were isolated from various sources such as urine, gastrointestinal tract samples, etc., varying from 0.6 % to 6.6 %.

These strains were recovered from patients aged 1 to 94 years old. The median age of the patients was 82 years, with patients over 60 years old accounting for 59.9 % (*n* = 100). As for patient gender, male was overwhelmingly higher than female (Table 2).

#### Antimicrobial susceptibility of all collected PA isolates

Overall, 58.7 % (*n* = 98) of the 167 clinical PA isolates were resistant to at least one tested drug. The resistant ratio of PA to the 13 tested antibiotics differed significantly from each other (*P* < 0.01), regardless of within a specific year or totally (Table 3). Among them, the highest resistant rates were found for PA against the TCC, IMP, LVX and MEM, reaching 41.9 %, 38.3 %, 35.3 % and 34.1 %, respectively. Tested PA strains were most sensitive to AMK and TOB, with fewer than 10 out of 167 strains found to be resistant. For antibacterial drugs other than the 6 types mentioned above, the resistant rate of PA strains ranged from 6.6 % to 26.9 %. The resistant rates of PA to PIP, ATM, IMP, MEM, CIP, and LVX varied significantly from year to year, whereas the resistant rates to the other 7 antibiotics remained relatively stable. However, there was no overall trend of change over the years, but a relatively high drug resistant rate was found in 2020. More isolates and longer duration of collection are needed for further confirmation of such trend.

**Table 3:** Number of resistant isolates within clinical *P. aeruginosa* isolates.

**Antibiotics**	**By year**				**By carbapenem resistance**			**Total (*n* = 167)**
	**2019 (*****n*** = **52)**	**2020 (*****n*** = **59)**	**2021 (*****n*** = **56)**	***P***	**CRPA**^a^ **(*****n*** = **70)**	**CNPA**^a^ **(*****n*** = **97)**	***P***	
**Penicillin**								
Piperacillin (PIP)	6	18	11	0.0475	28	7	<0.0001	35
**Cephalosporin**								
Ceftazidime (CAZ)	2	10	9	0.0723	17	4	0.0002	21
Cefepime (FEP)	4	12	5	0.0805	16	5	0.0008	21
**Monolactam**								
Aztreonam (ATM)	14	22	9	0.0374	32	13	<0.0001	45
**Carbapenem**								
Imipenem (IMP)	19	32	13	0.0027	64	0	<0.0001	64
Meropenem (MEM)	15	29	13	0.0085	57	0	<0.0001	57
β**-Lactam/** β**-Lactamase inhibitor**								
Ticarcillin/ clavulanic acid (TCC)	21	28	21	0.5372	47	23	<0.0001	70
Piperacillin/ tazobactam (TZP)	3	4	4	0.5969	8	3	0.0718	11
**Aminoglycoside**								
Amikacin (AMK)	0	3	2	0.2784	5	0	0.0119	5
Gentamicin (GEN)	3	10	4	0.0989	15	2	<0.0001	17
Tobramycin (TOB)	1	4	2	0.0910	5	2	0.1313	7
**Quinolone**								
Ciprofloxacin (CIP)	8	18	7	0.0336	21	12	0.0059	33
Levofloxacin (LVX)	20	28	11	0.0066	43	16	<0.0001	59
*P*	<0.0001	<0.0001	<0.0001	-	<0.0001	<0.0001	-	<0.0001

^a^CRPA (carbapenem-resistant *P. aeruginosa*) denoted isolates resistant to IMP or MEM or both; CNPA (carbapenem-nonresistant *P. aeruginosa*) denoted isolates other than CRPA.

It was particularly noteworthy that PA had developed a high level of resistance against carbapenems, one of the last resorts for effective treating of MDR gram-negative bacteria. Totally, 70 isolates were found to be resistant to imipenem and/or meropenem, accounting for 41.9 % of the tested strains. Among them, 51 were resistant to both carbapenems while 13 and 6 exhibited resistance against only IMP or MEM. Furthermore, compared to carbapenem-nonresistant PA (CNPA), the resistant rates to antibiotics other than TZP and TOB were significantly higher (*P* < 0.05) in CRPA (Table 3).

The combined use of β-lactamase inhibitor tazobactam, with PIP significantly enhanced its inhibitory effect against PA, leading to a decrease in the overall resistant rate from 21.0 % to 6.6 %. In contrast, another tested complex TCC with clavulanic acid as an inhibitor has no such effect. Seventy out of 167 isolates were found to be resistant to TCC. The proportion of strains with a MIC value over 128 μg/mL to ticarcillin (β-Lactam in TCC complex) was 46.7 % (*n* = 78), which was not significantly higher than that of TCC-resistant PA (with MIC value ≥128/2 μg/mL).

**Figure 1:**
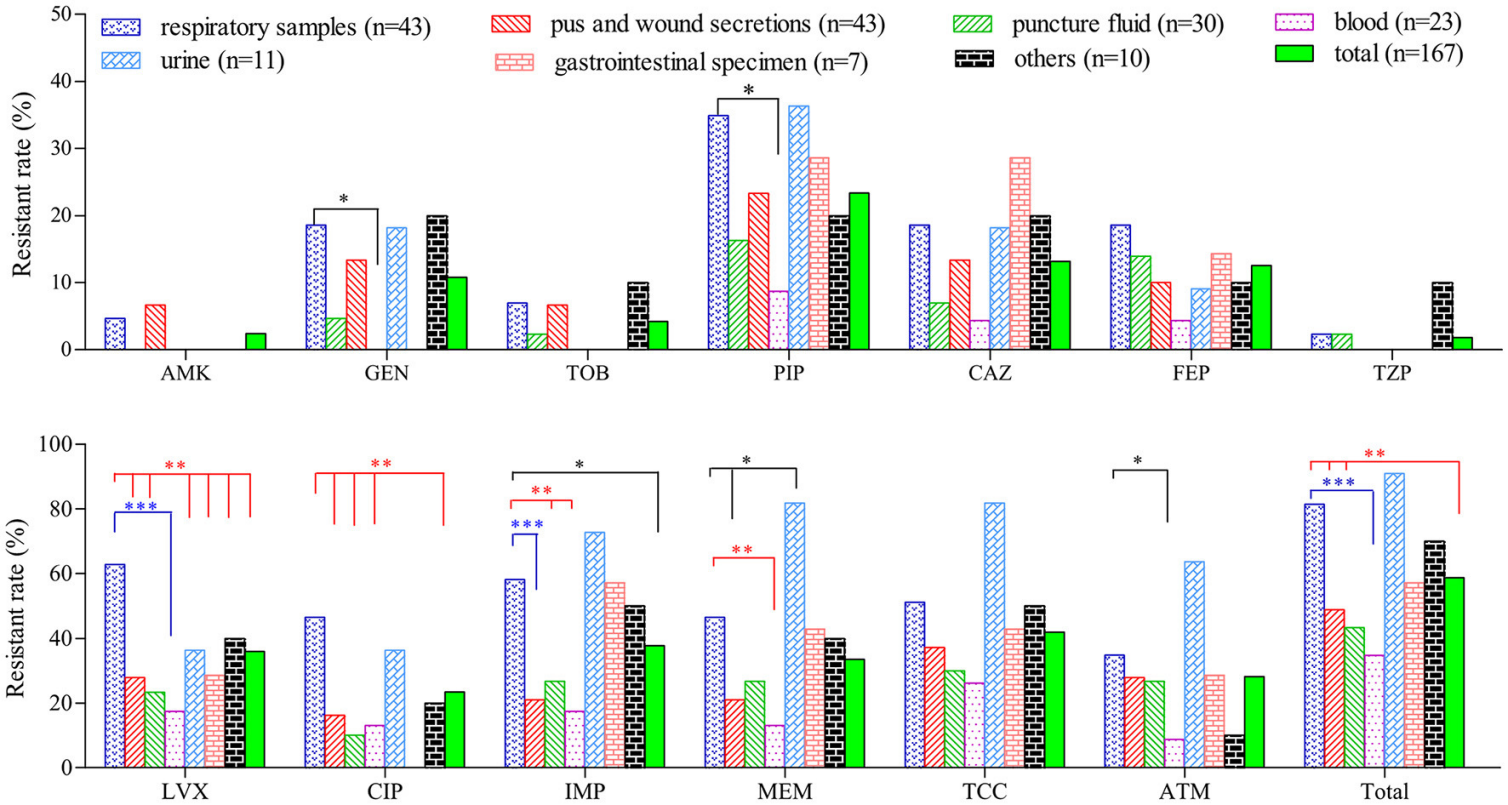
Comparison of resistant rates of *P. aeruginosa* isolated from different clinical specimens. ^*^(*P* < 0.05), ^**^(*P* < 0.01) and ^***^(*P* < 0.001) denoted statistical difference of resistant rate between PA strains of respiratory sources and those from other sources determined by chi-square test.

Compared with isolates collected from other clinical specimens, PA isolates from the respiratory tract (*n* = 43) and urine (*n* = 11) exhibited a higher resistant rate to the majority of the tested antibiotics (Figure 1). The proportion of resistant strains in these two sample types exceeded 80.0 %, which is significantly higher than strains from wound secretions, puncture fluids and blood. On contrary, strains collected from blood are highly sensitive to most tested drugs. Twenty-three blood isolates were fully sensitive to AMK, GEN, TOB and TZP while their resistant rates to the remaining 9 drugs were all below 20.0 %, except for TCC (26.1 %).

Ninety-eight resistant strains were distributed among 44 different resistotypes (Supplementary Table S1). Among them, 13 isolates were resistant to antimicrobial in one category, with 9 being carbapenem-resistant. Within the 23 isolates resistant to two types of antibiotic, there were 7 different resistotypes. The most common combinations were co-resistance to carbapenem with TCC or quinolone, and quinolone with TCC. An overwhelming majority (62 out of 98) of the resistant isolates were MDR. They were dispersed diversely into 34 different resistotypes, with co-resistance to quinolone-carbapenem- β-Lactam/β-Lactamase inhibitor-monolactam being the most common (*n* = 10). One isolate recovered from ascites of an 85 year old male patient in geriatric medicine department was found to resist against all 7 tested antimicrobial categories.

### Molecular epidemiology and phylogenetic relationship of CRPA isolates

**Figure 2:**
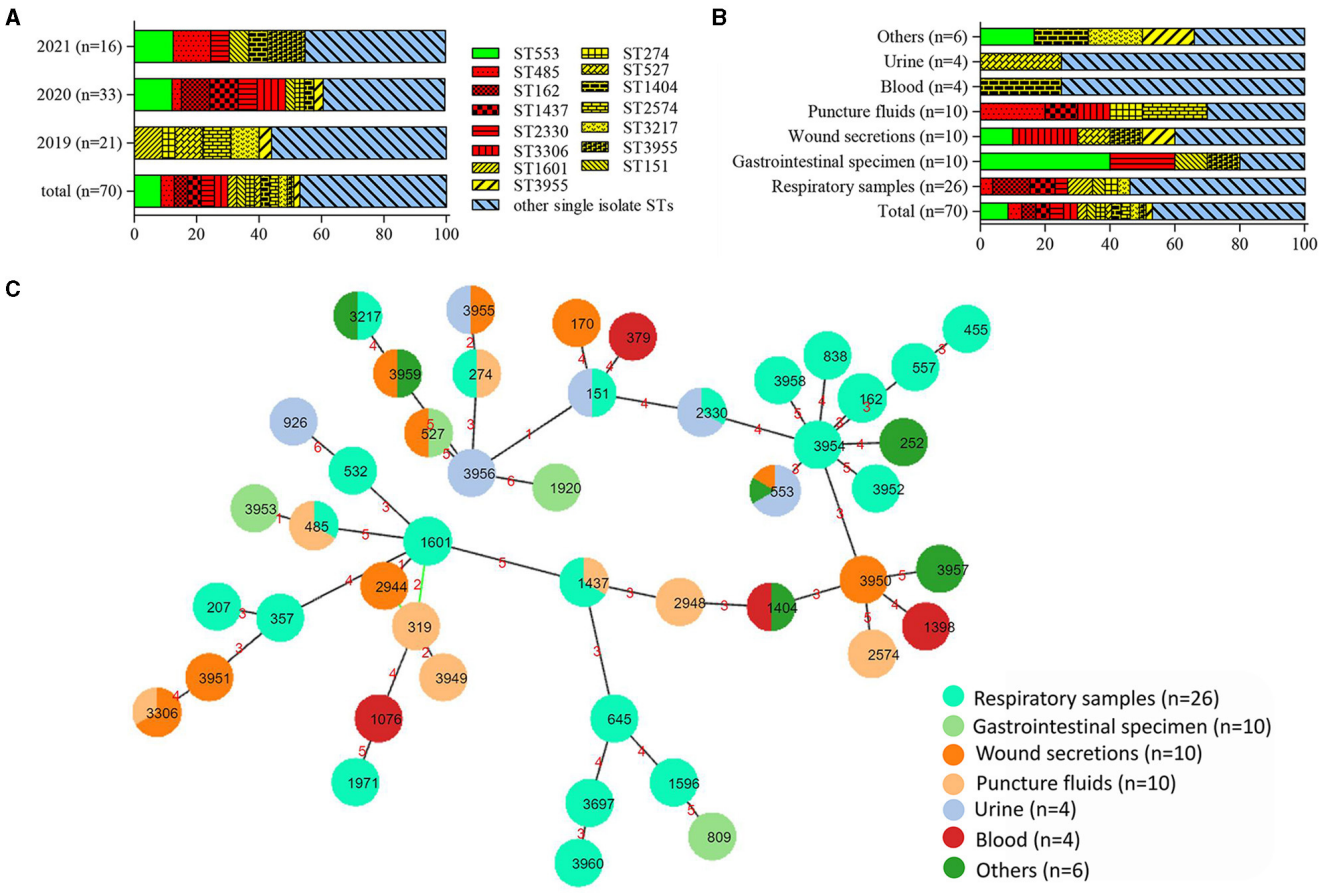
Distribution and relationship analysis of sequence types (STs) of 70 CRPA isolates. **(A)** Distribution of STs over different years. **(B)** Distribution of STs within various samples. Green, red, yellow and blue in **(A, B)** denoted STs with 6, 3, 2 and 1 isolates, respectively. **(C)** Minimum spanning tree created by PHYLOViZ Online (https://online.phyloviz.net/index). Each solid circle represented one ST and colors were assigned to nodes per sample sources. The circle size was related to the number of strains within this ST. The numbers on the lines connecting each node indicated the genetic distance between the two adjacent ST types.

High clonal diversity was revealed among 70 CRPA isolates, with 34 known sequence types (STs) identified among 56 isolates, while 12 new STs were found for 14 other isolates. ST553 was the most common ST with 6 strains identified. There are three strains in each of the five types: ST162, ST485, ST1437, ST2330, and ST3306. Out of the remaining 40 ST types, 9 types contain two strains, and 31 types only contain one isolate. The dominant ST types varied depending on the year of collection and sample types. Compared to the following 2 years, the isolates collected in 2019 were relatively diverse (Figure 2A). Among various sample sources, the PA isolates from respiratory samples exhibited the highest diversity in STs harboring more than 2 strains (Figure 2B). This result indicated that *P*. *aeruginosa* isolated from samples such as sputum and bronchoalveolar lavage fluid (BALF) were more diverse than those isolated from other samples. It was noteworthy that two previously reported high-risk STs, the regionally specific ST1971 (Zhao et al., 2023c) and the globally prevalent ST357 (Del Barrio-Tofiño et al., 2020), were found. They each only have one isolate collected from sputum and BALF, respectively.

The BURST analysis (https://pubmlst.org) revealed 4 CCs within the tested CRPA isolates. While the largest CC contained only 4 STs (ST1601, ST319, ST2944, and ST3949) with 5 strains, the remaining 3 CCs included only 2 STs. Out of a total of 46 ST types, thirty-six were found to be singletons. As showed in the MLST-based MST, four main groups were clustered, with most isolates from the respiratory specimen grouped in the center with a new ST (ST3954) (Figure 2C). Other CRPA isolates were relatively sporadically distributed and had distant genetic relationships.

**Figure 3:**
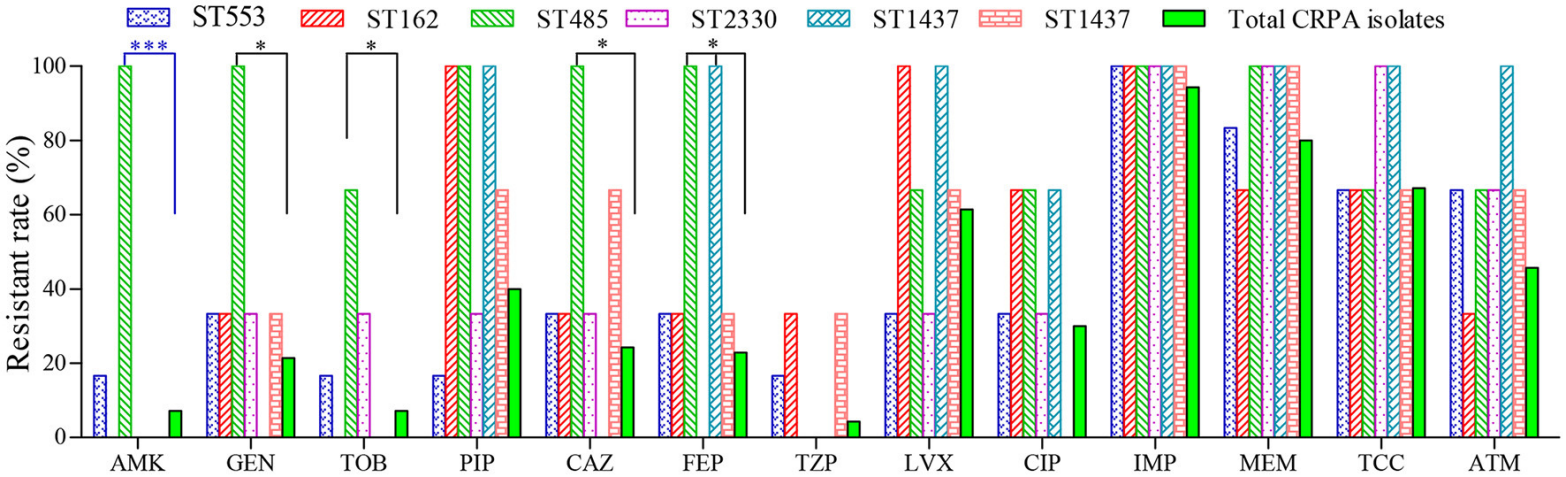
Antimicrobial susceptibility of CRPA strains belonging to the top six sequence types (STs). ^*^(*P* < 0.05) and ^***^(*P* < 0.001) indicated statistical differences determined by chi-square test.

Furthermore, no specific ST type was found to be associated with any particular resistance profile. Compared to other CRPA isolates, strains of ST485 were found to be more resistant against cephalosporins and aminoglycosides (Figure 3). However, it contained only 3 isolates in this study. Further investigation with more clinical isolates is needed to confirm this correlation.

### Carbapenem resistance mechanisms of CRPA isolates

#### Carbapenemase production by CRPA isolates

Using the mCIM, a total of 16 carbapenemase-positive isolates were identified from 70 CRPA strains, accounting for 22.9 %. As presented in Table 4, the specimen types and genetic distribution of such isolates were highly diverse. No correlation of carbapenem production with specific specimen type or ST could be found.

**Table 4:** Characteristics of 16 carbapenemase-positive CRPA isolates.

**No**.	**Sources**	**ST**	**MIC (**μ**g/mL)**		**Inhibitory zone diameter (mm)^a^**	**Carbapenemase genes**	**Relative transcription of Mex pumps**	**Biofilm**	**Mutation of porins** ^**b**^	
			**IMP**	**MEM**					**OprD**	**OpdP**
1	BALF	1601	16.0	4.0	7.3 ± 1.2	*bla*_IMP − 9_, *bla*_NDM − 1_, *bla*_VIM − 2_	5.3 ± 0.5	1.5 ± 0.2	Td(93)	S(15)
2	Blood	1076	16.0	8.0	6.3 ± 0.6	*bla*_IMP − 9_, *bla*_VIM − 2_	1.1 ± 0.2	2.6 ± 0.4	Tp(276)	S(15)
3	Live tissue sections	553	128.0	64.0	6.7 ± 1.2	*bla*_KPC − 2_, *bla*_IMP − 9_, *bla*_VIM − 2_	4.9 ± 0.5	1.7 ± 0.2	S(28)	S(16)
4	Wound	3951	64.0	64.0	6.0 ± 0.0	*bla*_NDM − 1_, *bla*_VIM − 2_	0.7 ± 0.08	3.9 ± 0.1	S(9)	S(15)
5	Sputum	485	32.0	16.0	6.0 ± 0.0	*bla*_IMP − 9_, *bla*_VIM − 2_	3.5 ± 0.5	1.4 ± 0.1	Tp(276)	S(16)
6	Purulent ear discharge	3957	2.0	16.0	6.0 ± 0.0	*bla*_IMP − 9_	4.9 ± 0.07	0.73 ± 0.1	N	Ti(336)
7	Gastric juice	1920	16.0	16.0	14.7 ± 1.2	*bla*_IMP − 9_	0.9 ± 0.05	0.22 ± 0.02	Td(134)	S(16)
8	Hydrothorax	3949	16.0	16.0	12.3 ± 1.5	*bla*_VIM − 2_	2.9 ± 0.2	3.8 ± 0.3	Td(68)	Td(6)
9	Blood	1398	64.0	32.0	11.3 ± 0.6	*bla*_IMP − 9_, *bla*_VIM − 2_	26.1 ± 0.9	1.3 ± 0.1	S(4)	S(16)
10	Wound	1404	64.0	32.0	11.7 ± 1.2	*bla*_IMP − 9_	4.1 ± 0.6	3.6 ± 0.4	Td(258)	Ti(336)
11	Sputum	1596	16.0	4.0	12.0 ± 1.0	*bla*_IMP − 9_	4.2 ± 0.4	1.4 ± 0.1	Tp(276)	Ti(336)
12	Wound	3306	32.0	32.0	14.7 ± 0.6	*bla*_KPC − 2_, *bla*_IMP − 9_	6.1 ± 0.9	2.7 ± 0.2	Ti(95)	S(16)
13	Wound	3306	64.0	16.0	14.7 ± 0.6	*bla*_KPC − 2_, *bla*_NDM − 1_, *bla*_VIM − 2_, *bla*_OXA − 23_	5.9 ± 0.6	2.2 ± 0.2	Ti(95)	S(16)
14	Ascites	3306	64.0	16.0	14.3 ± 1.2	*bla*_KPC − 2_, *bla*_VIM − 2_	2.2 ± 0.3	1.9 ± 0.2	Ti(95)	S(16)
15	Ascites	485	16.0	64.0	14.3 ± 0.6	*bla*_KPC − 2_, *bla*_IMP − 9_	0.8 ± 0.06	1.0 ± 0.1	Tp(276)	S(16)
16	Ascites	485	32.0	128.0	11.3 ± 1.2	*bla*_KPC − 2_, *bla*_IMP − 9_, *bla*_VIM − 2_	2.1 ± 0.3	1.9 ± 0.3	Tp(276)	S(16)

^a^Bactrial isolates with zone diameter of 6–15 mm or presence of pinpoint colonies within a 16–18 mm zone were defined as carbapenemase positive. ^b^T (number)-Truncated (length of the mutated porins) because of nucleotide insertion (Ti) or deletion (Td)-caused frameshift or nucleotide mutation-caused premature stop codon (Tp); S(number)-Substitution (number of substituted amino acids); N-No amplification.

Six out of the 16 strains exhibited strong enzyme activity comparable to that of the positive control strain *K. pneumoniae* ATCC BAA-1705. The meropenem disc treated with these CRPA strains showed little to no inhibitory effect against the carbapenem-susceptible indicator *E*. *coli* ATCC 25922 (Table 4). The enzyme activity of the remaining 10 strains was relatively weak, as indicated by an inhibitory diameter ranging from 11.3 to 14.7 mm. From the perspective of drug resistance spectrum, 13 out of 16 carbapenemase-producing CRPA (CP-CRPA) strains were resistant to both tested carbapenems, accounting for 81.3 %.

Through PCR screening, 16 CP-CRPA isolates were revealed to carry diverse carbapenemase genes (Table 4). MBL-encoding genes were the most widespread, with *bla*_IMP_ and *bla*_VIM_ detected in 12 (75.0 %) and 10 (62.5 %) isolates, respectively. However, *bla*_NDM_ is far less prevalent, being positive in only 3 out of 16 isolates. As for A-type carbapenemases, *bla*_KPC_ was detected in 6 isolates, while no *bla*_GES_ was amplified. D-type carbapenemases were the least common, with only 1 isolate carrying the *bla*_OXA − 23_ gene. Overall, 9 different carbapenemase gene profiles were found. Except for 5 isolates harboring a single *bla*_IMP_ (*n* = 4) or *bla*_VIM_ (*n* = 1) gene, most strains (68.8 %) were found to simultaneously carry two or more different carbapenemase genes. It was noteworthy that 6 isolates co-harbored MBLs along with class A (KPC-2) and/or class D (OXA-23-like) carbapenemases. Sequence analysis revealed that each identified gene encoded a distinct carbapenemase variant, such as IMP-9, NDM-1, VIM-2, and KPC-2.

Spearman correlation test revealed no significant (*P* > 0.05) association of carbapenemase activity (indicated by inhibitory zone diameter) with carbapenem resistance levels (indicated by MIC values of IMP or MEM) of CP-CRPA isolates. This may be due to the fact that different mechanisms interplayed with each, but not worked singularly, to determine the antibiotic phenotypes. For example, when *bla*_IMP − 9_ was first described, it seemed that MEM was consistently more active than IMP against *bla*_IMP − 9_-harboring strains (Xiong et al., 2006). However, the *bla*_IMP − 9_-carring isolate No. 6 in this study was found to be more resistant to MEM rather than IMP (Table 4). Other resistance mechanisms such as efflux overexpression by nearly 5 folds or OprD and OpdP mutation might also work there.

#### Mutation of carbapenem-specific porins OprD and OpdP in CRPA isolates

Sixty-eight out of 70 CRPA isolates showed at least one change in oprD. Forty-three isolates (61.4 %) were found to harbor truncated OprD proteins varying in length from 5 amino acids (aa) to 435aa instead of the intact 443aa. Among them, a frameshift mutation caused by the deletion or insertion of 1~26 bp nucleotides were most commonly found (n = 26). Premature stop codons created by point mutations were detected in 13 other isolates, with G831A leading to a truncated 276aa protein being most common (n = 7). Furthermore, the other 4 isolates simultaneously contained both forms of the mutation. For the remaining 27 isolates, 24 contained amino acid substitutions, 2 remained intact, and 1 was tested negative by PCR amplification, possibly due to a more severe mutation or complete deletion. Amino acid substitutions of T103S and F170L, located in loop 2 (L2) and loop 3 (L3) of the OprD topology, were most frequently found (8 out of 24). L2 and L3 in the OprD protein have been revealed to contain entrance and/or binding sites for imipenem (Ochs et al., 2000). Therefore, any mutations within these two regions could cause conformational changes and thus carbapenem resistance.

In contrast to OprD, the inactivated premature mutation in OpdP was less frequently encountered. Only 7 out of 70 CRPA strains contained premature OpdP due to the insertion or deletion of 1-2 nucleotides, resulting in truncated OprP with only 6aa or 336aa rather than 484aa. All other CRPA-sourced OpdP proteins were found to contain 15-21aa substitutions or a completely different sequence after amino acid 307. Among them, 11 kinds of amino acid mutations (N4Y, S9G, L17M, A19S, S20A, N31T, A34T, E42Q, A50E, T60S, and V63F) located within the first 63 amino acids were identified in the OpdP of all 63 isolates.

#### Biofilm formation by CRPA isolates

**Figure 4:**
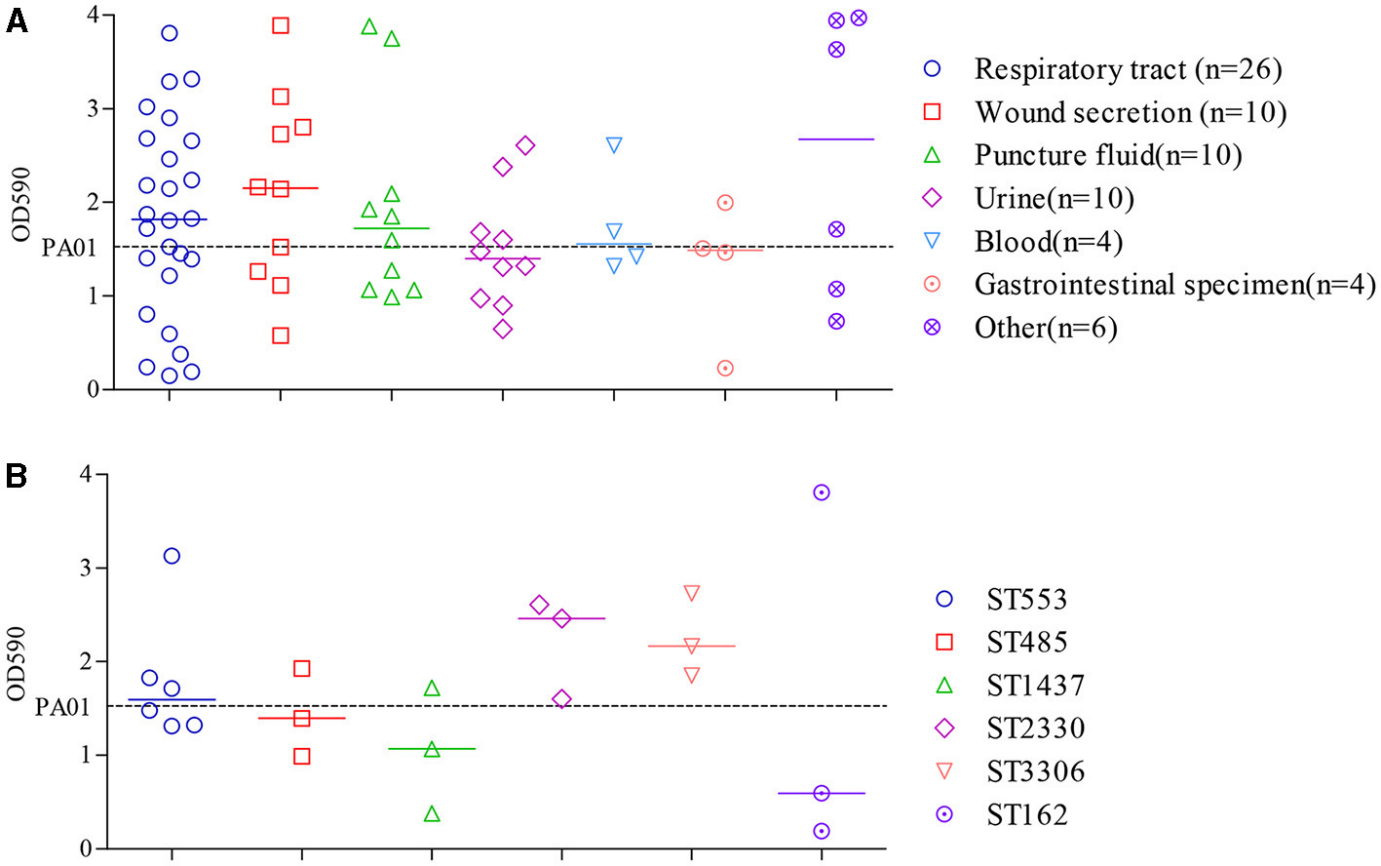
Comparison of biofilm formation by clinical CRPA isolates collected from various anatomical sites **(A)** or belonging to different MLST types **(B)**. Data points represented the average biofilm biomass of individual isolates as determined by the microtiter plate assay. Line bar represented the median biofilm biomass for each group. Only ST types with more than three individual strains were used for the comparison. One-way ANOVA analysis with the Holm-Sidak comparison test was used to determine statistical differences between groups. A dashed line represents the biofilm biomass formed by PAO1.

By conducting a one-way ANOVA, it was found that the biofilm developed by individual isolates significantly differed from each other (*P* < 0.0001). Among the 70 clinical CRPA strains assessed, 34 (48.6 %) and 14 (20.0 %) were found to exhibit significantly higher or statistically equivalent biofilm-forming capabilities compared to PAO1 (Figure 4A).

The median biofilm biomass (indicated by OD_590_) of *P*. *aeruginosa* strains from seven specimen types varied from 1.4 to 2.7. Except for isolates from urine and gastrointestinal specimens, which showed lower biofilm formation compared to PAO1, those from the other five sources exhibited a higher median level of biofilm formation. However, there were no statistically significant differences in biofilm formation ability among strains from different sources (*P* > 0.05).

ST types with more than three isolates were further assessed to determine the relationship between biofilm formation and specific ST. As shown in Figure 4B, clinical isolates from ST2330 and ST3306 exhibited a slightly higher, yet statistically insignificant, capacity for biofilm formation compared to the other four STs, as indicated by the median OD590 levels after crystal violet staining of the biofilms. Moreover, correlation analysis showed that the different ST types also did not have a significant impact on bacterial biofilm formation ability (*P* > 0.05).

#### Relative efflux pump gene transcript levels of CRPA isolates

Compared to PAO1, 72.9 % of CRPA isolates (51 out of 70) had higher general *Mex* (*MexA*~*MexY*) transcription levels, with median relative transcription level ranging from 1.1 to 149.2. For the other 19 isolates with lower *Mex* levels, the relative transcription level was 0.1~1.0 of that from PAO1. Therefore, most of the clinical CRPA isolates exhibited higher transcript levels of efflux pump genes compared to the control strain. One-way ANOVA analysis revealed that the total transcription level of the 8 tested *Mex* genes varied significantly among different isolates. However, no significant correlation (*P* < 0.05) was found between their overall transcriptional level and the sources of isolation.

**Figure 5:**
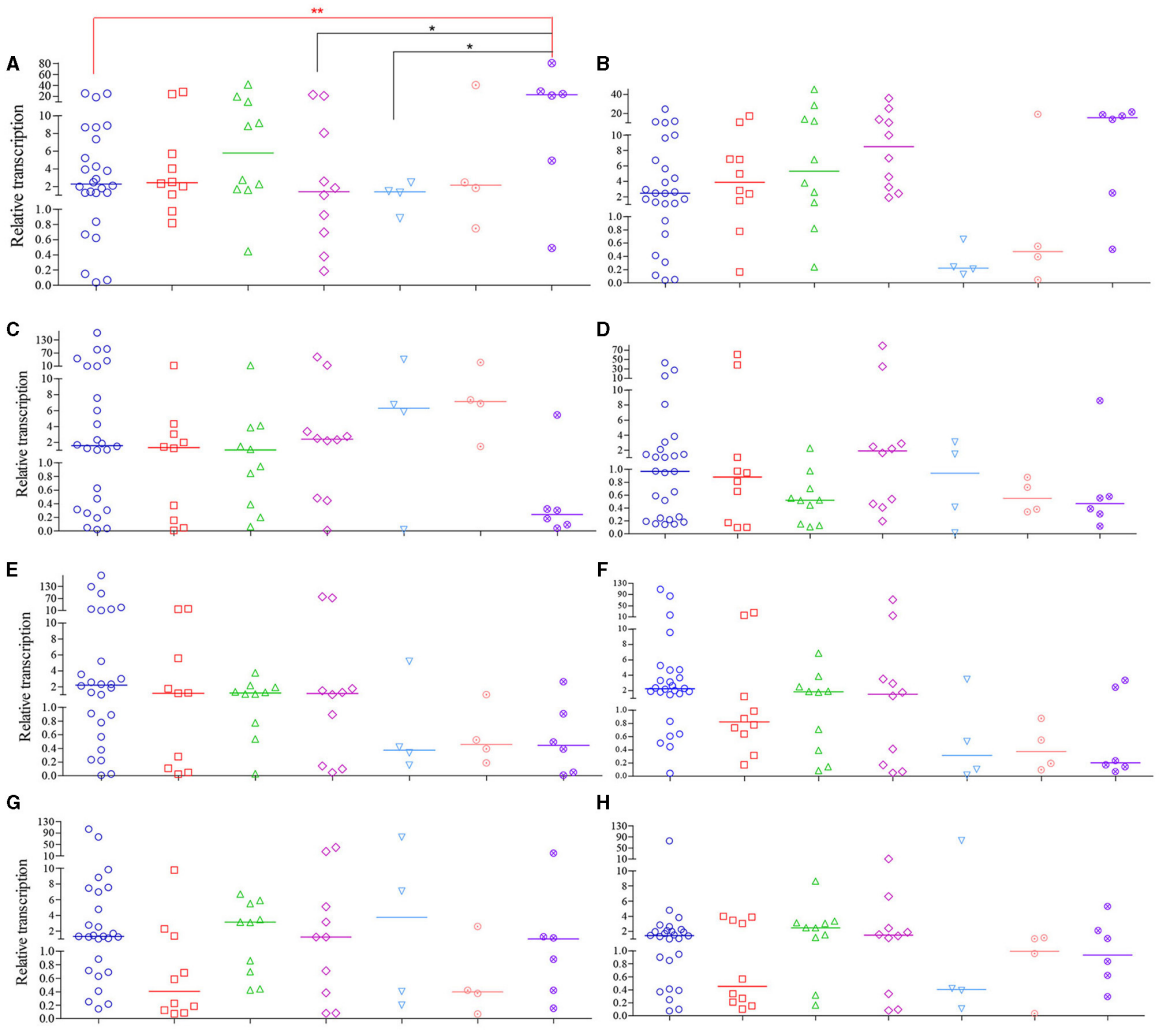
Relative transcription levels of *MexA*~*MexY* of CRPA isolates compared to PAO1. **(A–H)** *MexA, MexB, MexC, MexD, MexE, MexF, MexX* and *MexY*, respectively. The figure legends were the same as those of **(A)**. Data points represented the average transcription level of each gene from individual isolate compared to PAO1. Line bar represented the median transcription level for each group. One-way ANOVA analysis with Holm-Sidak comparison test was used to determine statistical differences between groups. ^*^(*P* < 0.05) and ^**^(*P* < 0.01) inidcated significant differences between the two groups as determined by chi-square test.

Among strains from various isolation sources, significant differences in relative transcription levels of specific *Mex* gene were observed only among the respiratory, urine, and blood samples of the *MexA* gene compared to the “others” group. In contrast, the transcription levels of the other seven genes were not significantly associated with the sample sources (Figure 5). Among them, only the median relative transcriptional levels of the *MexA* gene of PA isolates in all specimen types exceeded that of PAO1, while those of the *MexD* gene are lower than PAO1 in all types of samples except for those from urine. Regarding the overall transcriptional levels of each gene in 70 CRPA strains, apart from *MexD*, the transcriptional levels of all other genes were increased to varying degrees (1.1 to 2.9-fold) compared to PAO1.

#### Relationship of carbapenem resistance levels with the resistance-related mechanisms tested

No significant (*P* > 0.05) associations was found between carbapenem resistance levels (MIC values of CRPA against IMP or MEM) with any singular factor tested (i.e. carbapenemase activity or genotype, OprD or OpdP mutations, relative transcription of *mex* genes and biofilm production).

## Discussion

*P*. *aeruginosa* is one of the most common opportunistic pathogens in nosocomial infections, posing a significant threat to immunocompromised and ICU patients, with very high morbidity and mortality rates (Botelho et al., 2019; Behzadi et al., 2022a). In this study, we analyzed the clinical prevalence, epidemiology and carbapenem resistance mechanisms of 167 *P*. *aeruginosa* isolates collected from a tertiary hospital in southeast China between 2019 and 2021. It was found that respiratory and wound secretion samples were the primary specimen of isolation, while elderly male patients in the ICU and geriatric health units were the most common hosts. Nearly 60 % of the strains showed resistance to at least one antibiotic commonly used for treating *P*. *aeruginosa*, with 44 different resistotypes. The carbapenem-resistant isolates accounted for an alarmingly high rate of 41.9 % and were dispersed among 46 sequence types. A high proportion of porin mutation, together with elevated multidrug efflux pump gene transcription and biofilm production were found in CRPA isolates, while 22.9 % (*n* = 16) of them were carbapenemase-positive.

In this study, respiratory specimens were the most common sample where clinical PA isolates, especially CRPA, were recovered. Similar results were obtained not only from global cohort studies (Gill et al., 2022; Reyes et al., 2023), but also from specific national or regional research (Del Barrio-Tofiño et al., 2019; Folic et al., 2021; Zhao et al., 2023b; Bai et al., 2024). These facts showed that the respiratory system was the most common site of *P*. *aeruginosa* infection, making it a serious threat for hospital-acquired pneumonia. In terms of the demographics of the patients, elderly males in ICU or geriatric health units were found to be significantly more prevalent than other populations. The same epidemiological characteristics were also identified in other studies from Japan (Kainuma et al., 2018), the USA (Dunphy et al., 2021), China (Fan et al., 2016; Bai et al., 2024), and multi-center, multinational data (Gill et al., 2021; Reyes et al., 2023). The high incidence rate of male patients may be associated with the underlying lung diseases caused by their higher smoking rates compared to women.

The clinical PA isolates we collected exhibited a high resistant rate to various anti-pseudomonal drugs. The top 5 drugs with the highest resistant rates were TCC, IMP, LVX, MEM and ATM. The trend was consistent with the latest data reported by the China National Monitoring Network (CHINET) (available at http://www.chinets.com/Data/AntibioticDrugFast). The relatively higher resistant ratio of *P*. *aeruginosa* from respiratory specimen found in this work had also been documented in a nationwide study in Spain (Del Barrio-Tofiño et al., 2019) and a multidimensional surveillance in the USA (Dunphy et al., 2021). Through the collection of the 10 most commonly prescribed antimicrobials prior to PA isolation from each source, a strong association was found between specific antimicrobial resistance and the history of prescribing antipseudomonal medications in the latter study.

The CRPA ratio identified in this study was comparable to previous findings from a burn center in southwestern China (Yin et al., 2018) and a general hospital in Zhejiang (Hu et al., 2021), but twice of that from a hospital in southeastern Shanxi (Bai et al., 2024). However, the counterpart was reported to be alarmingly high (over 90 %) for 212 PA strains collected from Guangdong (Zhao et al., 2023a). Differences in carbapenem resistance were also notable within European Union (EU) member states, ranging from 2.4 % to 59.3 % in different countries (European Centre for Disease Prevention Control, 2023). Such differences may be attributed to variations in antimicrobial treatment strategies and methods for evaluating *in vitro* drug sensitivity among hospitals in different regions. Nationally, a resistant rate of 23.0 % (Zeng et al., 2023) was reported in China, equivalent to that reported in EU (European Centre for Disease Prevention Control, 2023) and United States (Tenover et al., 2022). Furthermore, we also found the CRPA isolates had a significantly higher resistant rate against various other antibiotics compared to the CNPA in this study. This may be due to the fact that carbapenem-resistant gram-negative bacteria usually carry resistance determinants to other drugs, thus limiting treatment options for their infections (Jean et al., 2022).

MLST is a crucial epidemiological typing method based on the sequences of seven different housekeeping genes. It has been widely utilized in studies on the evolution and population diversity of *P*. *aeruginosa* isolates (Castañeda-Montes et al., 2018). The high genetic heterogeneity of CRPA strains found in this research had also been reported in multiple regional (Hu et al., 2021; Zhao et al., 2023a,b; Bai et al., 2024) and national (Fan et al., 2016) studies in China. In addition, a similar phenomenon was found in an international collaborative study that involved strains from multiple countries (Reyes et al., 2023; Zhao et al., 2023c). The high genetic diversity and varied resistance patterns revealed among *P*. *aeruginosa* isolates collected in this study indicate that the clonal dissemination of this bacterium is low in this region. Contrastly, a nationwide survey in Spain reported a convergent relationship within1445 clinical PA isolates, with over 50 % of the strains being attributed to the top two most prevalent types (Del Barrio-Tofiño et al., 2019). Moreover, among the 46 STs revealed for 70 CRPA isolates in this study, two previously reported high-risk clones (HiRiCs), the global prevalent ST357 (Del Barrio-Tofiño et al., 2020) and China-specific ST1971 (Zhao et al., 2023b), were found. ST357 is an exoU+ T3SS genotype that appears to be particularly common in Asia, but has also been reported in Europe and South America (Kainuma et al., 2018). The multidrug-resistant and highly virulent clone ST1971 has only been identified in China and defined as a regional epidemic risk type for nosocomial healthcare (Zhao et al., 2023c). This is the first report of ST1971 in Fujian, which is geographically close to previously reported collection locations Guangxi and Guangdong.

Carbapenem-resistant *P*. *aeruginosa* poses a serious threat to public health due to the limited alternatives for antimicrobial therapy and high mortality rates (Mancuso et al., 2023). Carbapenemase production is thought to be a relatively uncommon but increasingly significant cause of carbapenem resistance in *P*. *aeruginosa* (Wang et al., 2021; Tenover et al., 2022). It had been revealed that the prevalence and carbapenemase enzymogram carried by CRPA varied widely across geographical regions. In our study, 22.9 % CRPA isolates were found to produce carbapenemase, genetically dominated by MBL. The proportion is higher than those reported from other researches in China. The carbapenemase gene-positive ratio in CRPA strains collected in Guangdong is 12.7 % (54/416), with *bla*_IMP − 45_ and *bla*_CARB − 3_ being the most prevalent (Zhao et al., 2023b). Only two out of the 57 CRPA collected in Shanxi, China carried the acquired carbapenemase genes, being *bla*_IMP − 1_- or *bla*_IMP − 10−_ and *bla*_OXA − 10_-positive, respectively (Bai et al., 2024). A national surveillance in China found 9.8 % of 182 IMP-insusceptible PA isolates carried KPC or IMP carbapenemases (Zhu et al., 2023). This was comparable to the data reported by Fan et al. (2016), in which 19 out of 254 (8.2 %) CRPA isolates collected nationally carried acquired carbapenemase genes. Similarly to our finding, IMP-9 was found to be most prevalent in this study, followed by VIM-2, IMP-1, IPM-10 and KPC-2. However, a high ratio of 32% (54 out of 171) were identified as CP-CRPA genetically for PA isolates collected from Zhejiang, Beijing, Shanghai, and Sichuan of China in a global cohort study (Reyes et al., 2023). KPC-2 (74.1 %) and VIM-2 (11.1 %) were most commonly found there. Globally, carbapenemase genes were detected in 22 % of 972 CRPA isolates, with 19% having a single carbapenemase gene and 3 % having two (Reyes et al., 2023). The prevalence of carbapenemase-positive CRPA (CP-CRPA) was lowest in the USA (2 %) and varied from 30 % to 69 % in other regions. According to another study on the global collection of CRPA with no Chinese isolates included, 33 % of the strains tested were positive for carbapenemase production, with rates varying by region from 11 % to 68 % (Gill et al., 2021). Among them, 86 % were genetically positive, with the most common being VIM followed by GES.

In contrast to the predominance of single enzymology reported in other studies (Gill et al., 2021, 2022; Reyes et al., 2023), we found that the majority (11 out of 16, 68.8 %) of CP-CRPA strains collected in this study co-harbored two or more carbapenemase genes. The coexistence of multiple enzymes further restricted treatment options. It has been proven that KPC-producing organisms are resistant to the anti-pseudomonal drug ceftolozane-tazobactam, while VIM carbapenemases cannot be inhibited by avibactam and relebactam, making them resistant to both ceftazidime-avibactam and imipenem-relebactam (Bail et al., 2022). On the other hand, the detection of carbapenemases, especially MBLs (Behzadi et al., 2020), will indicate the need to consider cefiderocol (Timsit et al., 2022; Gill et al., 2024) or combination therapy including aztreonam (Losito et al., 2022). Thus, the increased resistance of CP-CRPA contributed to the higher mortality reported among patients infected with these organisms compared to non-carbapenemase-producing CRPA (Reyes et al., 2023).

Except for carbapenemase, some other mechanisms have also been found to be critically responsible for carbapenem resistance in CRPA. In China, the inactivation of the oprD gene was commonly found in CRPA. Zhao et al. (2023b) showed oprD in an overwhelming proportion of 96.2 % of CRPA isolates were mutated. However, no correlation was explore between specific mutation forms with IMP or MEM resistant level. Overexpression of MexAB-oprM caused by mutations in regulatory genes *MexR* and *nalD* might also drive the development of CRPA. Similar results were also reported in a study covering 196 PA isolates collected in a burn center in southeast China (Yin et al., 2018). Mutational inactivation of oprD (88.65 %), accompanied by overexpression of AmpC (68.09 %), were both popular in CRPA. AmpC has been well characterized to related to resistance against penicillin, cephalosporins, β-lactamase inhibitors in *P*. *aeruginosa* (Berrazeg et al., 2015; Slater et al., 2020). The high proportion of elevated *AmpC* transcription revealed there may due to high resistant rate against AmpC-targeted drugs in CRPA isolates. Extended spectrum cephalosporinase-related carbapenem resistance was previously described in PA (Rodríguez-Martínez et al., 2009), but it is commonly considered as a rare carbapenem-resistance mechanism which was not explored in the present study. Furthermore, we also found a high mutation frequency of porins, being 97.1 % and 100 % for oprD and opdP, respectively. The outer membrane porins OprD and OpdP serve as important entry ports for carbapenems (Ude et al., 2021). The oprD mutations were also commonly found in a global collection of CRPA isolates. Reyes et al. (2023) found that 69 % of 972 CRPA isolates collected from 44 hospitals in 10 countries were identified to harbor oprD mutations. A high ratio of oprD mutations was also found in XDR PA isolates collected nationwide in Spain (Del Barrio-Tofiño et al., 2019). Regretfully, similar to Zhao et al. (2023b), we also cannot found a significant correlation of IMP or MEM MIC values with porin mutations. Moreover, the connection of antimicrobial resistance with biofilm production in CRPA isolates was also explored in this study, but again with negative results produced. Similar results were obtained by Gajdács et al. (2021) and Behzadi et al. (2022b) with clinical and environmental PA isolates, respectively. However, using MIC_90_ or MIC_50_ as indicator, Cho et al. (2018) revealed significantly higher resistance in aminoglycoside (AMK, GEN and TOB) and cephalosporins (CAZ and FEP), but not carbapenems (IMP and MEM) and quinolone (LVX) in biofilmproducer (*n* = 76) compared to non-producer (*n* = 6) within CRPA isolates. Likewise, a significantly elevated biofilm formation for XDR clinical PA group compared to nonXDR ones was reported by Kaiser et al. (2017). The difference may attribute to lacking of universal and robust association of these two factors among different PA isolates.

In conclusion, a high carbapenem resistance rate was found among clinical PA isolates collected from a specific hospital in southeast China. CRPA isolates there were most commonly recovered from respiratory specimens and were characterized with high genetic diversity. The emergence of high-risk clones (ST 357 and ST 1971) and prevalence of co-carriage of more than one carbapenemase genes in CRPA indicated an urgent need for specific antimicrobial therapy stewardship in this region. To the best of our knowledge, this is the first molecular epidemiological study on the clinical isolates of PA in this region. These findings highlighted the importance of regular monitoring and accurate establishing clinical diagnosis-based antibiotic prescriptions to improve the effectiveness of anti-infection measures and prevent the spread of CRPA. Unfortunately, though carbapenemase production, elevated biofilm production and multidrug efflux pump gene transcription, together with high proportion of carbapenem-related porin mutations were commonly found in CRPA isolates in this work, we failed to explore their significant association with the carbapenem resistance levels. This may be due to the following reasons: the carbapenem resistance of PA isolates was developed through mixed and complicated mechanisms and differed by strains. Multiple mechanisms interplayed with each other to finally produce the antimicrobial phenotype. So it may be hard to find a significant and robust association of the carbapenem MIC with singular and limited resistance-related mechanisms tested in this work. Furthermore, CRPA isolates is different from each other not only in varied carbapenem resistance levels, but also resistance to other antibiotics and maybe virulence levels and other phenotypes connecting to the tested mechanisms. Thereafter, we will seek coupled clinical isolates with similar genetic background but different carbapenem resistance and apply them for exploring the possible mechanisms underlying the carbapenem resistance development. More resistance-related mechanisms through next generation whole genomic sequencing, transcriptome and proteomics should be included for full exploration.

## Data Availability

The original contributions presented in the study are included in the article/Supplementary material, further inquiries can be directed to the corresponding authors.
