# Supplementary material for: Molecular epidemiology and carbapenem resistance mechanisms of Pseudomonas aeruginosa isolated from a hospital in Fujian, China
Source: Front Microbiol. 2024 Sep 5;15:1431154. doi: 10.3389/fmicb.2024.1431154 (PMC11410579; doi:10.3389/fmicb.2024.1431154)
Supplement: Supplementary file 1 [file Table_1.docx]

**TABLE S1 Antimicrobial resistant profile of 98 resistant *Pseudomonas aeruginosa* isolates**

| Types | Resistotypes | Specific resistant profiles | Number of isolates |
| --- | --- | --- | --- |
| SDR (resistant to a single antimicrobial category) | Carbapemem-R | IMP-MEM | 3 |
|  |  | IMP | 6 |
|  | Quinolone-R | LVX | 2 |
|  |  | LVX-CIP | 1 |
|  | β-Lactam/β-Lactamase inhibitor-R | TCC | 1 |
| DDR(resistant to two antimicrobial categories) | Carbapemem & β-Lactam/β-Lactamase inhibitor-R | IMP-MEM-TCC | 4 |
|  |  | MEM-TCC | 1 |
|  | Quinolone & Carbapemem-R | LVX-IMP | 2 |
|  |  | LVX-CIP-IMP | 2 |
|  |  | LVX-CIP-IMP-MEM | 1 |
|  | Quinolone & β-Lactam/β-Lactamase inhibitor-R | LVX-TCC | 1 |
|  |  | LVX-CIP-TCC | 4 |
|  | Monolactam & β-Lactam/β-Lactamase inhibitor-R | ATM-TCC | 4 |
|  | Aminoglycoside & Carbapemem-R | GEN-IMP-MEM | 2 |
|  | Aminoglycoside & Penicillin-R | GEN-PIP | 1 |
|  | Penicillin & β-Lactam/β-Lactamase inhibitor-R | PIP-TCC | 1 |
| MDR(resistant to ≥3 antimicrobial categories) | Quinolone & carbapenem & β-Lactam/β-Lactamase inhibitor & monolactam-R | LVX-IMP-MEM-TCC-ATM | 5 |
|  |  | LVX-CIP-IMP-MEM-TCC-ATM | 3 |
|  |  | LVX-IMP-MEM-TCC-TZP-ATM | 1 |
|  |  | LVX-MEM-TCC-ATM | 1 |
|  | Quinolone & penicillin & cephalosporin & carbapenem & β-Lactam/β-Lactamase inhibitor & monolactam-R | LVX-PIP-FEP-IMP-MEM-TCC-ATM | 3 |
|  |  | LVX-CIP-PIP-FEP-IMP-MEM-TCC-ATM | 2 |
|  |  | LVX-CIP-PIP-CAZ-FEP-IMP-MEM-TCC-TZP-ATM | 1 |
|  | Penicillin & cephalosporin & carbapenem & β-Lactam/β-Lactamase inhibitor & monolactam-R | PIP-CAZ-FEP-IMP-MEM-TCC-TZP-ATM | 4 |
|  | Quinolone & penicillin & cephalosporin & β-Lactam/β-Lactamase inhibitor & monolactam-R | LVX-CIP-PIP-FEP-TCC-ATM | 2 |
|  |  | LVX-CIP-PIP-FEP-TCC-TZP-ATM | 1 |
|  |  | LVX-CIP-PIP-CAZ-TCC-ATM | 1 |
|  | Quinolone & carbapenem & β-Lactam/β-Lactamase inhibitor-R | LVX-CIP-IMP-MEM-TCC | 2 |
|  |  | LVX-CIP-MEM-TCC | 1 |
|  |  | LVX-IMP-MEM-TCC | 1 |
|  | Quinolone & β-Lactam/β-Lactamase inhibitor & monolactam-R | LVX-TCC-ATM | 2 |
|  |  | CIP-TCC-ATM | 1 |
|  | Quinolone & penicillin & carbapenem & β-Lactam/β-Lactamase inhibitor & monolactam-R | LVX-PIP-IMP-MEM-TCC-ATM | 1 |
|  |  | LVX-PIP-MEM-TCC-ATM | 1 |
|  | Aminoglycoside & quinolone & carbapenem-R | AMK-CIP-IMP | 1 |
|  |  | GEN-LVX-CIP-IMP | 1 |
|  | Penicillin & cephalosporin & carbapenem & β-Lactam/β-Lactamase inhibitor-R | PIP-CAZ-FEP-IMP-MEM-TCC-TZP | 1 |
|  |  | PIP-CAZ-IMP-MEM-TCC | 1 |
|  | Aminoglycoside & quinolone & cephalosporin & carbapenem & monolactam-R | AMK-LVX-CIP-CAZ-FEP-IMP-MEM-ATM | 1 |
|  | Aminoglycoside & penicillin & cephalosporin & carbapenem & β-Lactam/β-Lactamase inhibitor-R | AMK-GEN-TOB-PIP-CAZ-FEP-IMP-MEM-TCC-TZP | 1 |
|  | cephalosporin & carbapenem & β-Lactam/β-Lactamase inhibitor-R | CAZ-IMP-MEM-TCC | 1 |
|  | Cephalosporin & carbapenem & β-Lactam/β-Lactamase inhibitor & monolactam-R | CAZ-IMP-MEM-TCC-ATM | 1 |
|  | Quinolone & cephalosporin & β-Lactam/β-Lactamase inhibitor & monolactam-R | CIP-FEP-TCC-ATM | 1 |
|  | Aminoglycoside & quinolone & penicillin & carbapenem & β-Lactam/β-Lactamase inhibitor & monolactam-R | GEN-LVX-CIP-FEP-PIP-IMP-MEM-TCC-ATM | 1 |
|  | Aminoglycoside & quinolone & carbapenem & monolactam-R | GEN-LVX-IMP-MEM-ATM | 1 |
|  | Aminoglycoside & quinolone & carbapenem & β-Lactam/β-Lactamase inhibitor-R | GEN-LVX-IMP-MEM-TCC | 1 |
|  | Aminoglycoside & quinolone & penicillin & cephalosporin & carbapenem & monolactam-R | GEN-LVX-PIP-CAZ-IMP-MEM-ATM | 1 |
|  | Aminoglycoside & quinolone & penicillin & carbapenem & β-Lactam/β-Lactamase inhibitor & monolactam-R | GEN-LVX-PIP-MEM-TCC-ATM | 1 |
|  | Aminoglycoside & penicillin & carbapenem-R | GEN-PIP-MEM | 1 |
|  | Aminoglycoside & quinolone & cephalosporin & carbapenem & β-Lactam/β-Lactamase inhibitor & monolactam-R | GEN-TOB-LVX-CIP-CAZ-IMP-MEM-TCC-ATM | 1 |
|  | Aminoglycoside & quinolone & carbapenem & β-Lactam/β-Lactamase inhibitor-R | GEN-TOB-LVX-CIP-IMP-MEM-TCC | 1 |
|  | Aminoglycoside & quinolone & penicillin & β-Lactam/β-Lactamase inhibitor-R | GEN-TOB-LVX-CIP-PIP-TCC | 1 |
|  | Aminoglycoside & quinolone & β-Lactam/β-Lactamase inhibitor & monolactam-R | GEN-TOB-LVX-CIP-TCC-ATM | 1 |
|  | Carbapenem & β-Lactam/β-Lactamase inhibitor & monolactam-R | IMP-MEM-TCC-ATM | 1 |
|  | Quinolone & penicillin & carbapenem & β-Lactam/β-Lactamase inhibitor & monolactam-R | LVX-CIP-PIP-IMP-MEM-TCC-ATM | 1 |
|  | Quinolone & penicillin & cephalosporin & β-Lactam/β-Lactamase inhibitor & monolactam-R | LVX-PIP-CAZ-FEP-TCC-ATM | 1 |
|  | Quinolone & penicillin & cephalosporin & carbapenem-R | LVX-PIP-CAZ-IMP | 1 |
|  | Quinolone & penicillin & cephalosporin & carbapenem & β-Lactam/β-Lactamase inhibitor-R | LVX-PIP-CAZ-IMP-MEM-TCC | 1 |
|  | Quinolone & penicillin & cephalosporin & β-Lactam/β-Lactamase inhibitor-R | LVX-PIP-CAZ-TCC | 1 |
|  | Penicillin & carbapenem & β-Lactam/β-Lactamase inhibitor-R | PIP-CAZ-TCC | 1 |
|  | Penicillin & cephalosporin & β-Lactam/β-Lactamase inhibitor-R | PIP-IMP-MEM-TCC | 1 |
|  | Aminoglycoside & quinolone & penicillin & cephalosporin & carbapenem & β-Lactam/β-Lactamase inhibitor-R | AMK-GEN-TOB-LVX-CIP-PIP-CAZ-FEP-IMP-MEM-TZP | 1 |
|  | All tested 7 antimicrobial categories-R | AMK-GEN-TOB-LVX-CIP-PIP-CAZ-FEP-IMP-MEM-TCC-TZP-ATM | 1 |
