# Supplementary figures and images for: Molecular epidemiology and carbapenem resistance mechanisms of Pseudomonas aeruginosa isolated from a hospital in Fujian, China
Source: Front Microbiol. 2024 Sep 5;15:1431154. doi: 10.3389/fmicb.2024.1431154 (PMC11410579; doi:10.3389/fmicb.2024.1431154)

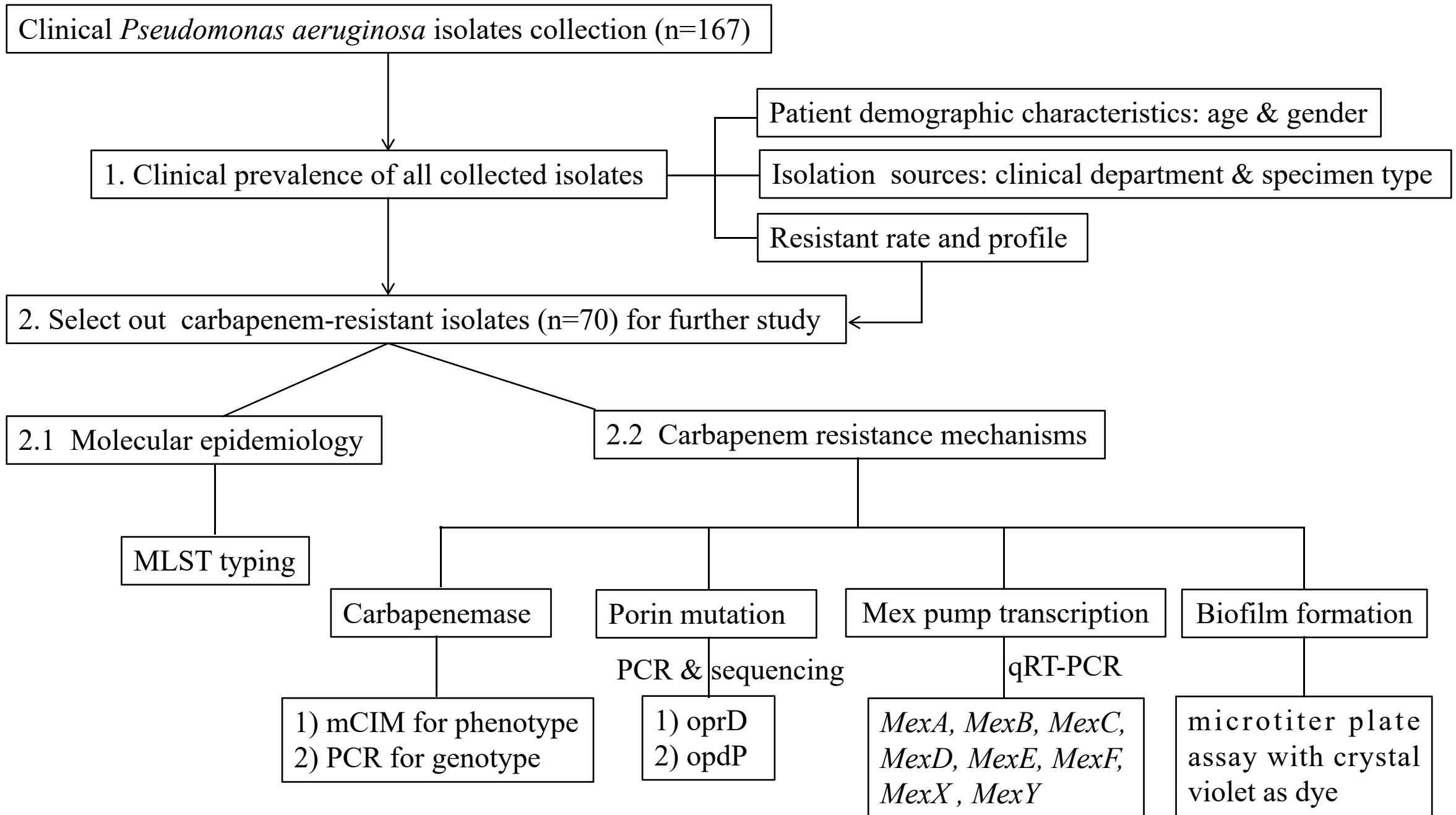

Supplement: Supplementary file 2 [file Image_1.pdf]
